# Congenital duodenal obstruction in the UK: a population-based study

**DOI:** 10.1136/archdischild-2019-317085

**Published:** 2019-06-22

**Authors:** George Stephen Bethell, Anna-May Long, Marian Knight, Nigel J Hall, On behalf of BAPS-CASS, Abigail Jones

**Affiliations:** 1 University Surgery Unit, Faculty of Medicine, University of Southampton, Southampton, UK; 2 National Perinatal Epidemiology Unit, Nuffield Department of Population Health, Oxford, UK; 3 Department of Paediatric Surgery, Cambridge University Hospitals NHS Foundation Trust, Cambridge, Cambridgeshire, UK

**Keywords:** neonatology, paediatric surgery, epidemiology, congenital Abnorm

## Abstract

**Objective:**

Congenital duodenal obstruction (CDO) comprising duodenal atresia or stenosis is a rare congenital anomaly requiring surgical correction in early life. Identification of variation in surgical and postoperative practice in previous studies has been limited by small sample sizes. This study aimed to prospectively estimate the incidence of CDO in the UK, and report current management strategies and short-term outcomes.

**Design:**

Prospective population-based, observational study for 12 months from March 2016.

**Setting:**

Specialist neonatal surgical units in the UK.

**Main outcome measures:**

Incidence of CDO, associated anomalies and short-term outcomes.

**Results:**

In total, 110 cases were identified and data forms were returned for 103 infants giving an estimated incidence of 1.22 cases per 10 000 (95% CI 1.01 to 1.49) live births. Overall, 59% of cases were suspected antenatally and associated anomalies were seen in 69%. Operative repair was carried out mostly by duodenoduodenostomy (76%) followed by duodenojejunostomy (15%). Postoperative feeding practice varied with 42% having a trans-anastomotic tube placed and 88% receiving parenteral nutrition. Re-operation rate related to the initial procedure was 3% within 28 days. Two infants died within 28 days of operation from unrelated causes.

**Conclusion:**

This population-based study of CDO has shown that the majority of infants have associated anomalies. There is variation in postoperative feeding strategies which represent opportunities to explore the effects of these on outcome and potentially standardise approach. Short-term outcomes are generally good.

**What is already known?**

- Congenital duodenal obstruction (CDO) is most commonly caused by duodenal atresia or duodenal stenosis which requires surgical repair usually within the first few days of life.
- Associated anomalies including cardiac and trisomy 21 are commonly associated with CDO.
- Postoperative feeding strategies include intravenous (parenteral) feed and feeding distal to the anastomosis with a trans-anastomotic tube, although neither have been shown to be superior.

**What this study adds?**

- The incidence of CDO in the UK is estimated at 1.22 cases per 10 000 (95% CI 1.01 to 1.49) live births.
- Sixty per cent of cases were suspected antenatally, yet only 46% of neonates were born at a surgical centre.
- There is wide variation in management of these infants, particularly regarding postoperative feeding strategy, which is not explained by demographic or clinical factors.
- Overall outcomes of CDO repair are good.

## Introduction

Congenital duodenal obstruction (CDO) caused by duodenal atresia or duodenal stenosis is a rare congenital anomaly. In the current era, approximately half of all cases are detected antenatally on fetal ultrasonography with the characteristic presence of a ‘double bubble’ sign in the upper abdomen representing dilated fluid-filled stomach and proximal duodenum. Antenatal suspicion of CDO should ideally prompt counselling by a paediatric surgeon and other genetic and structural abnormalities including chromosal anomalies should be considered since associated anomalies, including cardiac and aneuploidy, are common occurring in up to 84% of infants depending on the reported population.^1^ The definitive management of CDO is surgical, aiming to restore gastrointestinal continuity while avoiding damage to adjacent structures, principally the biliary and pancreatic ducts. A number of different procedures, techniques and approaches are used by surgeons, and none have proven benefit over any other.^2 3^

Following surgery, infants frequently have a period of upper gastrointestinal dysmotility secondary to a chronic in utero obstruction and proximal duodenal and gastric dilatation. A number of strategies are used to provide nutrition while normal gastrointestinal function returns including the use of intravenous (parenteral) feed and feeding into the bowel distal to the level of obstruction through a trans-anastomotic tube (TAT). No approach has been shown to be superior to any other.^4 5^

While overall outcome of infants born with CDO is good and surgical correction is usually curative, a number of comorbidities may affect outcome including trisomy 21 (Down’s syndrome), congenital cardiac disease and prematurity.^6^ The relative rarity of CDO makes single-centre studies inadequately powered to investigate the effects of these comorbidities. Similarly, accurate data on postoperative surgical outcomes are limited by small sample sizes.

## Aims of the study

The aims of this study were to estimate the incidence of CDO in the UK and to describe current management strategies, operative techniques and short-term outcomes.

## Methods

### Case definition and identification

**Figure 1:**
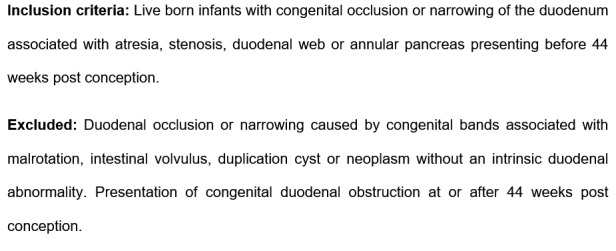
Case definition.

A predefined case definition was used (figure 1). Cases meeting this definition and presenting prior to a post-conceptual age of 44 completed weeks were prospectively identified over a 1-year period from 1 March 2016 at all 28 specialist paediatric surgical centres in the UK. The previously described British Association of Paediatric Surgeons Congenital Anomaly Surveillance System methodology was used.^7^

### Data collection

For each identified infant, a data collection form was completed at day 28 and 1 year following surgical repair. Forms were returned to the National Perinatal Epidemiological Unit, Oxford and entered into an electronic database. Cases not meeting the case definition and duplications were excluded.

### Statistical analysis

Descriptive analyses were undertaken using SPSS V.25. Live-birth data for 2016 were obtained for England and Wales, Scotland and Northern Ireland. Since monthly birth data were unavailable for all nations, yearly totals for 2016 were used to estimate the incidence of CDO. Data are median (range). Fisher’s exact, χ^2^ and Mann-Whitney U test were used as appropriate. A p value <0.05 was considered statistically significant.

To analyse weight change, LMSgrowth software (Royal College of Paediatrics and Child Health, UK) was used to generate weight-for-age z scores using British 1990 (UK 90) growth reference charts. Weight-for-age z score, also known as SD score, is a measure of the SD of weight from the mean value of a reference population matched for gestational age and sex.^8^

## Results

### Incidence

In total, 110 cases met the inclusion criteria during the study period, but for 7 cases, the 28-day data collection form was not returned so 103 (94%) infants were included in the study giving an incidence of 1.22 cases per 10 000 (95% CI 1.01 to 1.49) live births in the UK.

### Demographics

Demographic and clinical features are shown in table 1. There was a family history of CDO in three (3%) cases; two of these were individual parental cases and one was a sibling with duodenal atresia.

**Table 1:** Demographics and management overview

Male, n (%)	55 (53)
Gestational age at birth, weeks (range)	36 (26–42)
Birth weight, g (range)	2475 (800–4320)
Age at surgery, days (range)	2 (0–75)
Prenatal suspicion of CDO, n (%)	61 (59)
Preoperative upper GI contrast study, n (%)	35 (34)
Atresia type (figure 2), n (%)	
I	39 (38)
II	5 (5)
III	37 (36)
Not reported or not identified	22 (21)
Site of obstruction, n (%)	
Pre-ampullary	25 (24)
Post-ampullary	45 (44)
Not reported or not identified	33 (32)
Repair type, n (%)	
Duodenoduodenostomy	78 (76)
Duodenojejunostomy	15 (15)
Membrane resection	4 (4)
Duodenoplasty	3 (3)
Membrane incision	1 (1)
TAT placement, n (%)	43 (42)

All figures are given as median unless specified.

CDO, congenital duodenal obstruction; GI, gastrointestinal; TAT, trans-anastomotic tube.

**Figure 2:**
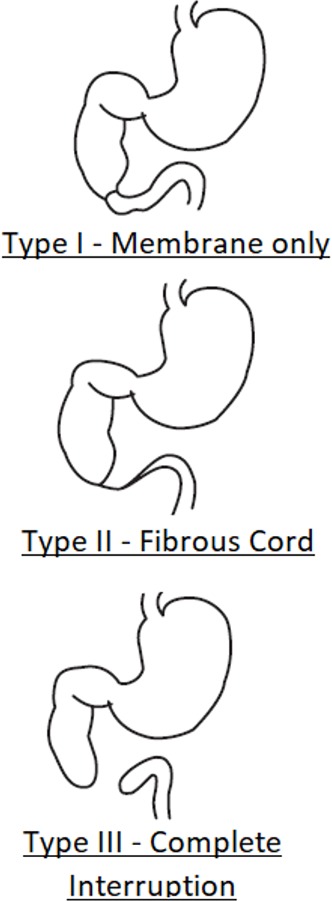
Type of atresia as defined in study protocol.

### Presentation and investigation

Postnatally, 56 (54%) infants were transferred to a paediatric surgical centre whereas the remainder were born in a unit with on-site paediatric surgical expertise. Of the 61 infants suspected to have CDO prenatally, 28 (46%) were transferred postnatally to a surgical centre. The median age of first review by a paediatric surgeon was 0 (0–13) days. A double bubble was seen on abdominal X-ray in 85 (83%) of cases and the majority of infants proceeded to surgical repair on the basis of this radiological finding. However, an upper gastrointestinal (GI) contrast study was undertaken in 35 (34%) neonates. Indications for requesting this modality of imaging were diagnostic uncertainty on plain radiology alone (n=23), bilious vomiting (n=9), non-bilious vomiting (n=3) and difficulty feeding (n=2).

### Associated anomalies

Associated anomalies were seen in 71 (69%) infants and are shown in table 2. The most frequently associated anomalies were cardiac, seen overall in 48%.

**Table 2:** Associated anomalies with CDO (n=71*)

Associated cardiac anomaly	49 (48)
Isolated PDA	6 (6)
PDA with other structural cardiac anomaly	19 (19)
VSD	17(17)
PFO	15(15)
ASD	10(10)
AVSD	4 (4)
Tetralogy of Fallot	3 (3)
Coarctation of aorta/hypoplasia	2 (2)
Other	11 (11)
Annular pancreas	13 (13)
Biliary tree anomaly	1 (1)
Abnormal rotation	22 (21)
Other gastrointestinal anomaly	16 (16)
Anorectal malformation	6 (6)
OA with TOF	5 (5)
Isolated OA	4 (4)
Meckel’s diverticulum	2 (2)
Ileal atresia	2 (2)
Cloaca anomaly	1 (1)
Hirschsprung’s disease	0 (0)
Genetic/chromosomal anomaly	38 (37)
Trisomy 21	33 (32)
Other	5 (5)
Other structural anomalies	16 (16)
Renal	5 (5)
Limb	3 (3)
Spine	2 (2)
Other	11 (11)

Data are n(%).

*Note that infants may have multiple anomalies; therefore, figures add up to more than 100%.

ASD, atrial septal defect; AVSD, atrioventricular septal defect; OA, oesophageal atresia; PDA, patent ductus arteriosus; PFO, patent foramen ovale; TOF, tracheo-oesophageal fistula; VSD, ventricular septal defect.

### Surgical management

One infant died prior to repair of CDO due to other anomalies. The remainder all underwent surgical repair at median age 2 (0–75) days. The infant who underwent surgery at day 75 of life was extremely preterm at birth and had significant morbidity related to prematurity that required stabilisation prior to repair of CDO. There was no difference in age at operation between those born at less than and those more than 1500 g and those born before and after 36 completed weeks of gestation (2 vs 3 days (p=0.38) and 2 vs 3 days (p=0.48), respectively).

The majority of repairs were undertaken via laparotomy (97/102, 95%), with only five (5%) having attempted laparoscopic repairs (four completed successfully and one converted to laparotomy as the infant was unable to tolerate pneumoperitoneum). Operative repair performed is shown in table 1. There was formal assessment of intestinal rotation in 89 (87%) cases which identified 22 (22%) cases of abnormal rotation leading to formal surgical correction in 18 (82%) infants. There was one intraoperative complication reported—a duodenal tear during initial repair which was treated with closure and an omental patch.

At the time of the initial operative intervention, a trans-anastomotic feeding tube was placed in 43 (42%) cases and a tunnelled central venous catheter (CVC) in 18 (18%) instances. There was no statistically significant difference in birth weight (p=0.78), gestational age at birth (p=0.88), the presence of a prenatal diagnosis of CDO (p=0.16) or presence of associated anomalies (p=0.83) in those that had TAT insertion compared with those who did not have a TAT inserted (table 3). However, those who had a TAT placed underwent surgery at a younger age (2 (0–14) vs 4 (0–75) days, p=0.005) than those without a TAT. When those who had CVC insertion were compared with those who did not (table 4), there was no statistically significant difference in birth weight (p=0.22), gestational age at birth (p=0.99), prenatal diagnosis of CDO (p=1.0), age at initial surgery (p=0.76) or presence of associated anomalies (p=0.26).

**Table 3:** Group characteristics of TAT vs no TAT

	TAT (n=43)	No TAT (n=59)	P value
Birth weight, g (range)	2350 (800–4320)	2500 (830–3755)	0.78
Gestational age at birth, weeks (range)	36.3 (27.0–39.9)	36.3 (25.6–42.3)	0.88
Prenatal CDO diagnosis, n (%)	29 (67)	31 (53)	0.16
Associated anomalies present, n (%)	29 (67)	41 (69)	0.83
Age at surgery, days (range)	2 (0–14)	4 (0–75)	**0.005**

CDO, congenital duodenal obstruction; TAT, trans-anastomotic tube.

**Table 4:** Group characteristics of CVC/PICC vs no CVC/PICC

	CVC/PICC (n=91)	No CVC/PICC (n=11)	P value
Birth weight, g (range)	2475 (800–4320)	2350 (1840–4080)	0.22
Gestational age at birth, weeks (range)	36.3 (25.6–42.3)	36.3 (27.0–39.1)	0.99
Prenatal CDO diagnosis, n (%)	54 (59)	6 (55)	1.00
Associated anomalies present, n (%)	62 (68)	8 (73)	0.26
Age at surgery, days (range)	3 (0–75)	2 (0–6)	0.76

CDO, congenital duodenal obstruction; CVC, central venous catheter; PICC, peripherally inserted central venous catheter.

### Other procedures performed at the same time

Excluding CVC insertion, there were 38 (37%) operative procedures carried out in 27 infants at the same time as surgery for CDO repair, predominantly for treatment of associated congenital anomalies (table 5).

**Table 5:** Additional procedures undertaken (n=38)

Ladd’s procedure*	13 (13)
Colostomy formation†	5 (5)
Appendicectomy (with normal rotation)	4 (4)
Tracheo-oesophageal fistula ligation	4 (4)
Gastrostomy formation	3 (3)
Distal ileal atresia repair	2 (2)
Resection of Meckel’s diverticulum	2 (2)
Anoplasty	1 (1)
Oesophagogastroduodenoscopy	1 (1)
Oesophageal atresia repair	1 (1)
Oesophagostomy	1 (1)
Chest drain insertion	1 (1)

Additional procedures undertaken at time of CDO repair in 27 infants.

Data are n(%)

*Correction of malrotation with/without appendicectomy.

†4 anorectal malformations and one cloaca.

### Postoperative management and outcomes

Postoperatively, an upper GI contrast study was undertaken in 12 (12%) infants due to suspicion of either an anastomotic stricture (n=5), anastomotic leak (n=3) or other indication (n=4). Findings were functional anastomotic obstruction (n=1) and leak from an oversewn distal tracheo-oesophageal fistula in an infant whose oesophageal atresia was not amenable to primary repair (n=1). The remaining 10 postoperative contrast studies were normal.

Postoperative complications related to CDO repair were wound infection (n=3), wound dehiscence (n=1), incisional hernia (n=1), functional obstruction treated with duodenoplasty (n=1) and leak from a duodenoduodenal anastomosis (n=1). In addition, one infant had a laparotomy for a suspected leak which was not proven. In total, three (3%) infants underwent repeat laparotomy within 28 days for reasons related to CDO.

Median time to starting enteral feeds was 4 (1–35) days and median time to achieving full enteral feeds was 12 (2–44) days. Parenteral nutrition (PN) was used in 90 (88%) infants with a median duration of 11 (2–86) days. Peripherally inserted central venous catheters or tunnelled CVCs were used in 91 (89%) infants with a median of 1 (0–8) catheters per infant. CVC-related complications occurred in 23 CVCs (18%) affecting 21 infants (21%). Only two infants were managed with neither a TAT nor PN.

Median postoperative hospital stay was 20 (6–73) days and 20 (25%) infants were still in hospital at 28 days after operation (data available in 81 cases). There were two (2%) deaths within 28 days of operative intervention due to causes unrelated to CDO, both of whom had trisomy 21. Therefore, the majority of infants and all of those with no associated anomaly survived to 28 days. Mean change in weight-for-age z score was −0.95 (SD 0.65) from birth to 28 days.

## Discussion

To our knowledge, this is the largest prospective population-based study reporting incidence, management and early outcomes of infants with CDO. Despite the majority of infants having an associated anomaly requiring surgical intervention, mortality is low and is in keeping with rates reported previously.^1^

Incidence of CDO in this study was 1.22 cases per 10 000 (95% CI 1.01 to 1.49) live births. Other studies have estimated this figure to be between 0.83 (95% CI 0.56 to 1.17) and 1.54 (95% CI 1.14 to 1.94).^1 9^ Two of these explored the incidence of CDO across Europe and therefore included part of the geographical population in the current study.^10 11^ The English National Congenital Anomaly and Rare Disease Registration Service collects data on congenital abnormalities detected in England including CDO, and in 2016 reported a prevalence of CDO of 1.5 (95% CI 1.2 to 2) per 10 000 total births.^12^ Unfortunately, historical figures are not available in the study population to compare changes, if any, over time.

With advances in prenatal fetal ultrasonography, more cases of congenital anomalies such as CDO are detected prior to delivery permitting antenatal counselling and delivery at a surgical centre. In this series, 59% of cases (n=61) were suspected prenatally yet just 47 (46%) cases were born in a centre with on-site neonatal surgical expertise. This discrepancy may reflect unanticipated onset of labour but more likely represents the lack of colocation of maternity and neonatal surgical services. Of note, a number of specialist children’s hospitals in the UK are not colocated with maternity services necessitating postnatal transfer of a newborn infant. The overall prenatal detection rate of 59% is similar to the 53% reported in a single-centre study from the USA^13^ and 52% from a European prenatal ultrasound population-based study.^14^ Consequently, in nearly half of all infants with CDO, there will be no prenatal detection. This emphasises the importance of considering CDO as a possible diagnosis in any infant with clinical signs of upper GI tract obstruction in the initial hours of life.

It is widely reported that associated structural and chromosomal anomalies are both common in CDO.^9 11 15^ In this study, over half of all infants with CDO had one or more coexisting congenital anomaly. In keeping with the existing literature, about one-third had trisomy 21. The most common associated abnormalities are cardiac to the extent that almost half of all infants with CDO have a congenital cardiac abnormality. We therefore recommend that all infants with CDO undergo echocardiography before hospital discharge. Of particular surgical relevance, 10% of infants also had oesophageal atresia, but just 2% had a distal intestinal atresia.

In 2001, the first laparoscopic repair of CDO was reported,^16^ yet it is interesting that the laparoscopic approach to CDO repair in the UK is infrequently undertaken. We suspect this reflects an absence of evidence to support a specific benefit of laparoscopy for CDO repair. A systematic review which compared 67 laparoscopic repairs and 113 open repairs found similar outcomes in each group except for a longer operative duration with laparoscopic repair.^17^ On closer inspection, anastomotic complications consisting of stenosis, strictures and leaks were seen in 4.4% of the laparoscopic group versus 1.8% of the open group. In this current study, there were only two anastomotic complications of which one occurred following laparoscopic repair.

This study demonstrates wide variation in practice both intraoperatively and postoperatively in infants with CDO. The largest area of variation in care for CDO appears to be method of feeding in the postoperative period. TAT feeding is reported to reduce the need for PN, decrease time to full enteral feeds and also reduce the cost of feeding.^4 18^ However, in this study, only 42% of infants had a TAT placed for feeding and the majority of these infants also had a CVC placed and received PN. It would therefore appear that the feeding plan varies between individual clinicians and that some infants receive PN when they could have achieved adequate feeding outcomes with just TAT feeding. The drivers behind this variation, and in particular whether there are underlying surgeon or institutional preferences, or clinical features related to the infant, are unclear. Despite this being a national study, we do not have sufficient statistical power to examine surgeon or institutional preferences reliably due to the low incidence of the condition.

A strength of our study is the use of proven surveillance methodology in order to capture all cases. A further strength is a high data return rate (94%). Data were collected prospectively over a short time period, therefore representing contemporary practice when compared with other series that have collected data over longer.^19 20^ Detailed clinical and surgical data have been reported by the actual clinicians treating the infant. Despite a relatively large sample, this study remains limited in its ability to detect rare complications. For instance, there was no mortality related to CDO and its management in this series although this outcome has been reported by others.^15^ Finally, our outcomes here are limited to 28 days following decision for surgical intervention; we plan to report longer-term outcomes once they are available.

## Conclusion

This national population-based study has provided a population-based estimate of incidence in the UK and shown variation in surgical technique and wider variation in postoperative feeding strategies. Short-term outcomes in these infants are generally excellent. Future work is required to explore whether feeding strategies influence outcomes, since if they do, a uniform approach should be adopted to the postoperative care in CDO.
